# Polysaccharides from *Enteromorpha prolifera* Improve Glucose Metabolism in Diabetic Rats

**DOI:** 10.1155/2015/675201

**Published:** 2015-08-10

**Authors:** Wenting Lin, Wenxiang Wang, Dongdong Liao, Damiao Chen, Pingping Zhu, Guoxi Cai, Aoyagi Kiyoshi

**Affiliations:** ^1^Department of Nutrition and Health Care, School of Public Health, Fujian Medical University, Fuzhou, Fujian 350108, China; ^2^Department of Health Inspection and Quarantine, School of Public Health, Fujian Medical University, Fuzhou, Fujian 350108, China; ^3^Fujian Province Key Laboratory of Environment and Health, School of Public Health, Fujian Medical University, Fuzhou, Fujian 350108, China; ^4^Institute of Tropical Medicine, Nagasaki University, Nagasaki 852-8523, Japan; ^5^Nagasaki Prefectural Institute of Environmental Research and Public Health, Nagasaki 2-1306-11, Japan; ^6^Department of Public Health, Nagasaki University Graduate School of Biomedical Sciences, Nagasaki 852-8523, Japan

## Abstract

This study investigated the effects of polysaccharides from *Enteromorpha prolifera* (PEP) on glucose metabolism in a rat model of diabetes mellitus (DM). PEP (0, 150, 300, and 600 mg/kg) was administered intragastrically to rats for four weeks. After treatment, fasting blood glucose (FBG) and insulin (INS) levels were measured, and the insulin sensitivity index (ISI) was calculated. The morphopathological changes in the pancreas were observed. Serum samples were collected to measure the oxidant-antioxidant status. The mRNA expression levels of glucokinase (GCK) and insulin receptor (InsR) in liver tissue and glucose transporter type 4 (GLUT-4) and adiponectin (APN) in adipose tissue were determined. Compared with the model group, the FBG and INS levels were lower, the ISI was higher, and the number of islet *β*-cells was significantly increased in all the PEP groups. In the medium- and high-dose PEP groups, MDA levels decreased, and the enzymatic activities of SOD and GSH-Px increased. The mRNA expression of InsR and GCK increased in all the PEP groups; APN mRNA expression increased in the high-dose PEP group, and GLUT-4 mRNA expression increased in adipose tissue. These findings suggest that PEP is a potential therapeutic agent that can be utilized to treat DM.

## 1. Introduction

Diabetes mellitus (DM) is characterized by endocrine-induced metabolic disorders with complex etiology [1]. The major pathophysiological issue for DM patients is insufficient insulin (INS) secretion associated with the destruction of pancreatic islet *β*-cells and insulin resistance (IR), ultimately resulting in complications in numerous systems, such as the cardiovascular and renal systems [2, 3]. DM-associated cardiovascular complications have the highest disability and mortality rates and have caused the greatest damage of all the chronic complications in China [4]. Until now, DM patients had to consistently receive injections of exogenous INS or take oral hypoglycemic drugs, and this long-term treatment induces drug side effects and creates a significant economic burden. Therefore, it is worth exploring diet composition and nutritional status to prevent and/or identify therapies to treat this chronic disease, as it is seldom studied.

Polysaccharides, which belong to a class of essential organic compounds that form the base of all living creatures, are generated by combining multiple monosaccharides of the same type or of different types [5]. With advancements in related research, the activity of marine algal polysaccharides has become a hot research topic worldwide [6, 7]. Marine algae are rich in polysaccharides, which account for more than 50% of their dry weight. Studies have demonstrated that marine algal polysaccharides are predominantly sulfated polysaccharides [8]; the antioxidant activity of sulfated glucans is greater than that of regular glucans [9]. Previous studies have shown that many marine algal polysaccharides, including laminarin 13, exopolysaccharide of* Porphyridium cruentum* 18,* Spirulina* polysaccharides 22, and alginic sodium diester 25, play a role in lowering blood sugar and treating DM complications, such as hyperlipidemia [10–13]. However, different marine algal polysaccharides contain different functional agents; therefore, they may work to prevent or treat DM by different mechanisms and further studies in this area are warranted.

*Enteromorpha prolifera* belongs to the phylum* Chlorophyta*, class* Chlorophyceae*, order Ulvales, and genus* Enteromorpha*. It is a large, natural wild green alga that grows in offshore shoals and is widely distributed in rock pools in river mouths and tidal zones.* Enteromorpha prolifera* has been recognized as an edible and medicinal alga since ancient times. It is highly abundant, and currently, the majority grows and dies without human management or control.* Enteromorpha prolifera* naturally has a strong reproductive capacity, which can have a significant negative impact on the environment and aquaculture [14]. The large-scale outbreak of large green algae, such as* Enteromorpha prolifera,* is now called “green tide,” which is considered to be a type of marine disaster similar to red tide [15]. However, if these algae could be utilized, then waste could be turned into a valuable resource.* Enteromorpha prolifera* polysaccharides (PEP) are soluble sulfated heteropolysaccharides. Previous studies have demonstrated that PEP have numerous functional bioactivities, such as strong antibacterial, anti-inflammatory, antitumor, and antioxidant functions, as well as the abilities to reinforce immunity and regulate blood lipid levels [16–19]. However, whether PEP has an anti-DM pharmacological effect has been seldom studied.

Based on these observations, the purpose of this study was to determine the anti-DM effects of PEP in a rat model of DM and investigate the possible underlying mechanisms. Our study provides experimental evidence for the clinical treatment of DM and the theoretical basis for the development and application of PEP.

## 2. Material and Methods

### 2.1. Animals

Ninety six-week-old male Sprague-Dawley (SD) rats weighting 90 ± 10 g were obtained from the Shanghai SLAC Laboratory Animal Co. LTD. The experimental protocol was approved by the Animal Care and Use Committee of Fujian Medical University. The experimental procedures were carried out in accordance with international guidelines for the care and use of laboratory animals.

### 2.2. High-Sugar, High-Fat Diet

A high-sugar, high-fat diet was prepared based on the following formula that was slightly modified from a previous study [20]: 65% basal feed, 20% sucrose, 10% lard, 2.5% egg yolk powder, and 2.5% cholesterol. The basal feed was prepared and provided by the Laboratory Animal Center of Fujian Medical University; the main components were 30% corn, 21% soybean cake, 10% bran, 28% wheat middlings, 9% fish powder, 1.7% CaHPO_4_, 0.3% salt, 1.5% yeast, and 1% fish liver oil.

### 2.3. Chemicals

PEP was extracted and prepared in our laboratory from* Enteromorpha prolifera* that was collected from the coastal region of Fujian, China, using the water extraction-alcohol precipitation method. The obtained PEP sample was a soluble polysaccharide. Streptozotocin (STZ) was obtained from Sigma Chemical Co. (St. Louis, MO, USA). All other reagents were of analytical grade.

### 2.4. Experimental Design

After one week of acclimatization, the rats were randomly divided into the control group of ten rats and model groups of eighty rats. Then, the control group was fed a normal diet, and the model group was fed the above high-sugar, high-fat diet for four weeks. After four weeks, the model group was injected intraperitoneally with 30 mg/kg streptozotocin (STZ) [21]; after 72 h, plasma glucose levels were determined, and a glucose concentration ≥200 mg/dL indicated that the hyperglycemic rat (DM) model is established [22]. Subsequently, 50 successful DM model rats were divided into five groups of ten rats each: the model, metformin hydrochloride (metformin HC), and low-, medium-, and high-dose PEP groups. The metformin HC group received 500 mg/kg metformin hydrochloride intragastrically, and the low-, medium-, and high-dose PEP groups were dosed intragastrically with PEP at 150 mg/kg, 300 mg/kg, and 600 mg/kg (distilled water as the solvent), respectively, once per day. The model and control groups received equivalent distilled water intragastrically. The doses of PEP used in this study were selected based on our previous studies and in accordance with other previous reports [23, 24]. The intervention period was four weeks. At the end of the intervention, the rats were fasted for 12 hours and then sacrificed by decapitation, and the blood samples, perirenal white adipose tissue, liver, and pancreatic tissue samples were harvested and stored at −80°C until use.

### 2.5. Characterization of Polysaccharides from* Enteromorpha prolifera*

DEAE-cellulose and Sephadex-100 were used to purify and separate the PEP. The structural characteristics of PEP were observed using infrared spectrum analysis. The compositions of the PEP monosaccharides were determined by gas chromatographic analysis.

### 2.6. Fasting Blood Glucose (FBG) Measurements and the Oral Glucose Tolerance Test (OGTT)

After the rats were fasted for 12 hours, the blood sample was collected and FBG was determined on days 0, 7, 14, 21, and 28 after the intervention treatment using a blood glucose meter on the caudal vein [25]. After measuring the FBG on day 28, the rats in each group were dosed intragastrically with a 50% glucose solution (2 g/kg); the blood glucose levels after 0.5, 1, and 2 h were measured for the OGTT [26].

### 2.7. Fasting Insulin (FINS) Measurements and the Insulin Sensitivity Index (ISI)

After the rats were fasted for 12 hours, the blood sample was collected and the serum FINS levels for all rats in each group were measured using the ELISA method. The ISI is the reciprocal of the product derived from the multiplication of the FBG and INS levels. Because this index demonstrated a skewed distribution, the natural logarithm was used: ISI = Ln 1/(FBG × FINS) [27].

### 2.8. Determination of Serum Malondialdehyde (MDA), Super Oxide Dismutase (SOD), and Glutathione Peroxidase (GSH-Px)

Malondialdehyde (MDA) content was assayed using the TBA method of Buege and Aust [28]. SOD activity was measured using the method of Misra and Fridovich [29]. Glutathione peroxidase (GPH-PX) activity was assayed using the method of Rotruck et al. [30]. These biomolecules were quantified using test kits that were obtained from the Nanjing Jiancheng Bioengineering Institute (Nanjing, China).

### 2.9. Histopathological Examination of the Pancreas

Pancreatic tissues were fixed in a 10% neutral formalin solution for 24 h. After being washed overnight with running water, the samples were embedded in paraffin, sectioned, baked, and stained with hematoxylin and eosin (H&E). A specific antibody against insulin/proinsulin was utilized to observe the residual *β*-cells in islets by using immunohistochemical method (IHC). The *β*-cells in islets were calculated as integrated optical density per area (mean integrated optical density) using the Image-Pro Plus 4.5 image analysis software.

### 2.10. Detecting the mRNA Expression of Related Genes in Adipose and Liver Tissue

The mRNA levels of GCK, InsR, GLUT-4, and APN were determined using real-time PCR. Total RNA was extracted from each sample (ten samples per group) using the RNAiso Plus reagent (TaKaRa Biotechnology Co., Ltd., Dalian, China) according to the manufacturer's instructions. RNA was reverse-transcribed into cDNA using the PrimeScript Reverse Transcriptase Reagent (TaKaRa Biotechnology Co., Ltd., Dalian, China) according to the manufacturer's instructions. Briefly, the following substrates were placed into a tube: 2.0 *μ*g of RNA in 2 *μ*L, 1.0 *μ*L of 50 *μ*M oligo-dT primer, 1.0 *μ*L of PrimeScript RT Enzyme Mix, 1.0 *μ*L of 100 *μ*M random hexamers, 5.0 *μ*L of 5 x PrimeScript Buffer, and 10.0 *μ*L of RNase-free dH_2_O, for a total volume of 20 *μ*L. The mixture was incubated at 37°C for 15 min and at 85°C for 5 sec. After reverse transcription, the samples were stored at −20°C until polymerase chain reaction (PCR) was performed.

Real-time fluorescent quantitative PCR was carried out with the SYBR Green I fluorescent dye reagent (SYBR Premix Ex Taq II, TaKaRa Biotechnology Co., Ltd.) and an ABI System Sequence Detector 7500 (Applied Biosystems Inc., USA). *β*-actin was used as an internal standard to normalize all samples for potential variations in mRNA content. Amplifications were performed under the following conditions: Stage 1, 95°C for 30 sec, 1 cycle; Stage 2, 95°C for 10 sec and 60°C for 34 sec, 40 cycles. The relative expression levels of GCK, InsR, GLUT-4, and APN were normalized by the internal standard *β*-actin and expressed as a percentage of the *β*-actin value. The results are given as the mean ± standard deviation. The oligonucleotide sequences of primers used for detecting the mRNA expression of these markers are presented below:

 
*β*-actin
   Forward Primer:
   5′-GGAGATTACTGCCCTGGCTCCTA-3′
   Reverse Primer:
   5′-GACTCATCGTACTCCTGCTTGCTG-3′
 GCK
   Forward Primer:
   5′-AGTATGACCGGATGGTGGATGAA-3′
   Reverse Primer:
   5′-CCAGCTTAAGCAGCACAAGTCGTA-3′
 InsR
   Forward Primer:
   5′-TCATGGATGGAGGCTATCTGGA-3′
   Reverse Primer:
   5′-TCCTTGAGCAGGTTGACGATTTC-3′
 GLUT-4
   Forward Primer:
   5′-CCGGGACACTATACCCTATTCA-3′
   Reverse Primer:
   5′-AGGACCAGTGTCCCAGTCACTC-3′
 APN
   Forward Primer:
   5′-CTGTCTGTACGAGTGCCAGTGGA-3′
   Reverse Primer:
   5′-CTTCATGACTGGGCAGGATTAAGAG-3′

### 2.11. Statistical Analysis

All data are expressed as the mean ± standard deviation. The IBM SPSS 19.0 software was used to analyze the data. Differences among the groups were compared by a one-way ANOVA. An LSD test was used to further analyze the differences between the two groups if equal variances were assumed. If equal variances were not assumed, Dunnett's C test was used instead. A *P* value of less than 0.05 was considered significant.

## 3. Results

### 3.1. Characterization of Polysaccharides from* Enteromorpha prolifera*

**Figure 1:**
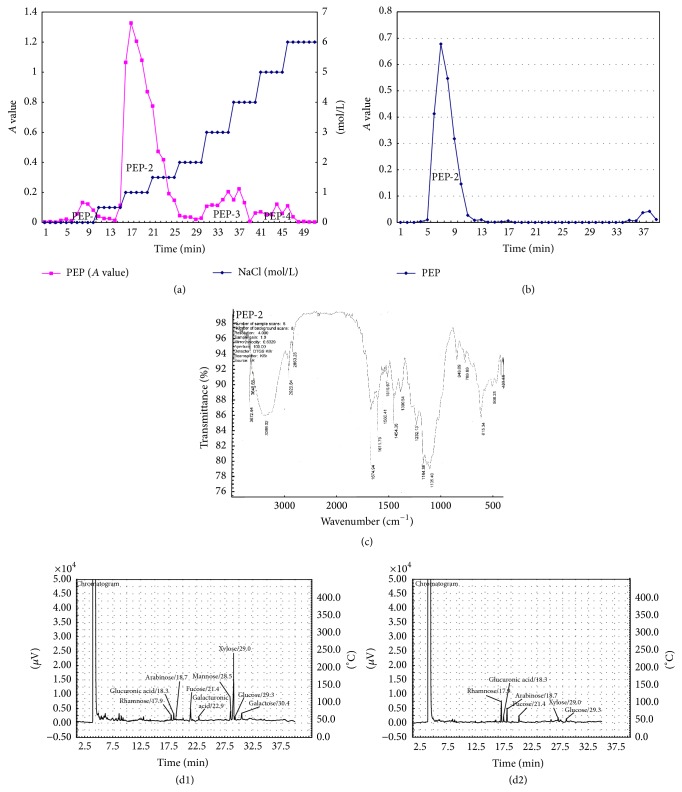
Characterization of polysaccharides from* Enteromorpha prolifera*. (a) shows four peaks in the eluting curve, PEP-1, PEP-2, PEP-3, and PEP-4. The peak area of PEP-2 is the largest and contains 161 mg of polysaccharides. (b) shows the basic graph of PEP-2, which has a single, symmetrical elution peak. (c) shows the wavenumbers of 3386.02 cm^−1^ as the stretching vibration of O–H, 2923.84 cm^−1^, 2853.28 cm^−1^, and 1454.36 cm^−1^ as the vibration of** –**CH2–. The 1390.54 cm^−1^ and 1232.13 cm^−1^ symmetric stretching vibrations corresponded to two S=O groups of sulfate groups. Furthermore, 848.09 cm^−1^ and 769.89 cm^−1^ are the antisymmetric and symmetric stretching vibrations of the sulfate groups C–O and S–O. (d1) shows that the chromatogram of different standard monosaccharides. (d2) shows that PEP-2 is mainly composed of rhamnose, glucuronic acid, arabinose, fucose, xylose, and glucose.

PEP was separated to four components, PEP-1, PEP-2, PEP-3, and PEP-4, by DEAE-cellulose methods (Figure 1(a)). PEP2 is the major component, at up to 81%. PEP-2 shows a single symmetric peak when it flows through Sephadex-100 (Figure 1(b)). Infrared spectrum analysis shows that PEP-2 is a sulfate ester-containing polysaccharide (Figure 1(c)). Gas chromatographic analysis shows that the monosaccharides of PEP-2 were rhamnose, glucuronic acid, arabinose, fucose, xylose, and glucose (Figures 1(d1) and 1(d2)). The proportions of the monosaccharides are 5.12 : 1.32 : 3.38 : 1.62 : 1 : 1.03, respectively.

### 3.2. The Effect of PEP on Body Weight, Food Consumption, and Drinking Water Intake in DM Rats

During the experiment, gut dysfunction and death were not observed in DM rats. However, body weight and daily food consumption were significantly lower in the model group compared with the control group (*P* < 0.05). On the contrary, the daily drinking water intake was significantly higher than that in the control group (*P* < 0.05). Compared with the model group, drinking water intakes were lower in the medium- and high-dose PEP groups (*P* < 0.05). Body weight and food consumption were not significantly different between the low-, medium-, and high-dose PEP groups, the metformin HC group, and the model group (*P* > 0.05) (Table 1).

### 3.3. The Effects of PEP on FBG and OGTT in DM Rats

**Figure 2:**
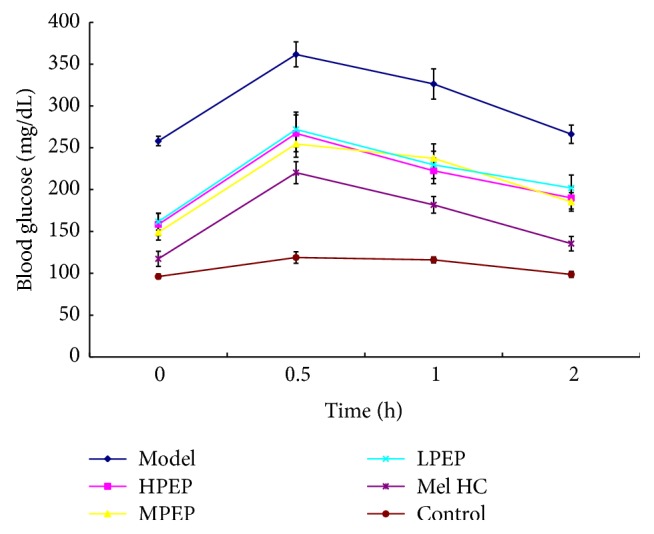
The effect of PEP on OGTT in DM rats. A DM model was established by feeding rats a high-fat diet and injecting them with a low dose of STZ. After the model was established, PEP (0, 150, 300, and 600 mg/kg) was administered intragastrically as an intervention for 28 d (*n* = 10 rats). Subsequently, the OGTT was performed. Control: control group; Model: model group; Mel HC: metformin HC group; LPEP: low-dose PEP group; MPEP: medium-dose PEP group; HPEP: high-dose PEP group.

Before the intragastric treatment (on day 0), the FBG was significantly higher in the model, metformin HC, and PEP groups than in the control group (*P* < 0.05). One week after the intragastric intervention, the FBG levels decreased in all groups; however, from weeks 1 through 4, rats in the model group had a higher FBG than rats in the other groups. After four weeks of intragastric treatment, the FBG was lower in the PEP and metformin HC groups than in the model group (*P* < 0.05). Compared with day 0, the FBG levels in the low-, medium-, and high-dose PEP groups and in the metformin HC group decreased (*P* < 0.05) (Table 2). The OGTT indicated that the blood glucose levels in all the rats peaked 30 min after glucose injection and essentially recovered within 2 h. Compared with the model group, the blood glucose levels recovered faster in the PEP and metformin HC groups, but the differences between the PEP groups were not significant (Figure 2).

### 3.4. The Effect of PEP on Serum FINS Levels and the ISI in DM Rats

At the end of the experiment, FINS levels were higher and the ISI was significantly lower in the model group compared with the control group (*P* < 0.05). Compared with the model group, FINS levels were lower in the high-dose PEP and the metformin HC groups (*P* < 0.05). The ISI was lower in the low-, medium-, and high-dose PEP and metformin HC groups than in the model group (*P* < 0.05) (Table 3).

### 3.5. The Effect of PEP on Serum MDA Levels and the Enzymatic Activity of SOD and GSH-Px in DM Rats

Compared with the control group, the MDA levels were higher and the GSH-Px activity was lower in the model group (*P* < 0.05); although there were no significant differences in SOD enzymatic activity (*P* > 0.05), it exhibited a decreasing trend. Compared with the model group, the MDA levels in the low-, medium-, and high-dose PEP and metformin HC groups were lower (*P* < 0.05). The SOD enzymatic activity in the low-, medium-, and high-dose PEP groups increased. The GSH-Px activity increased in the medium- and high-dose PEP and metformin HC groups (Table 4).

### 3.6. The Effect of PEP on the Histopathological Changes in the Pancreas in DM Rats

**Figure 3:**
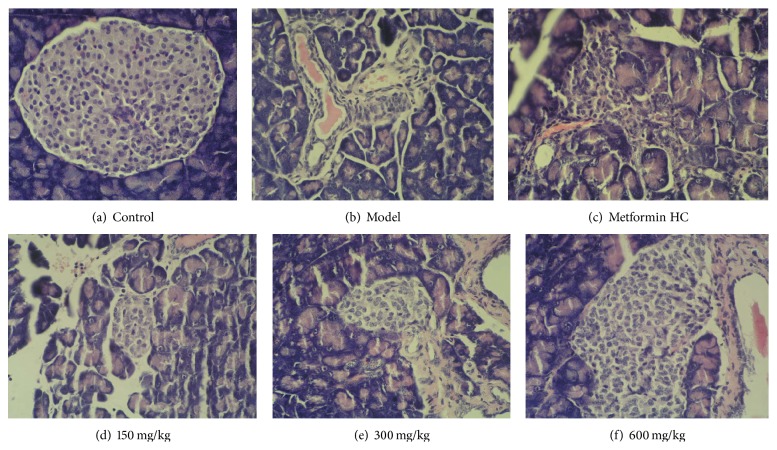
The effect of PEP on the histopathological structure of the pancreas in DM rats (H&E staining). A DM model was established by feeding rats a high-fat diet and injecting them with a low dose of STZ. After the model was established, PEP (0, 150, 300, and 600 mg/kg) was administered intragastrically as an intervention for 28 d (*n* = 10 rats). The pancreatic tissues were collected, embedded, sectioned, and stained with H&E. (a) control: control group; (b) model: model group; (c) metformin HC: metformin HC group; (d) 150 mg/kg: low-dose PEP group; (e) 300 mg/kg: medium-dose PEP group; (f) 600 mg/kg: high-dose PEP group.

Light microscopy following H&E staining indicated that the pancreatic acini and islets were intact in the control group. In the model group, the islets were scattered, the islet volume was significantly decreased, most islet cells were atrophic and more lightly stained, there were fewer cells, the cytoplasm was significantly decreased, the nuclei exhibited large-scale condensation, and lymphocytic filtration was extensive around and invading into the islets. In the metformin HC group, the morphological changes were not as severe as those in the model group. In the medium- and high-dose PEP groups, the number of islets and *β*-cells was significantly increased, the morphology and structure were essentially normal, the cytoplasm was homogenous and in greater quantity, the nucleoli were clear, and occasionally a small amount of lymphocytic infiltration was observed (Figure 3).

**Figure 4:**
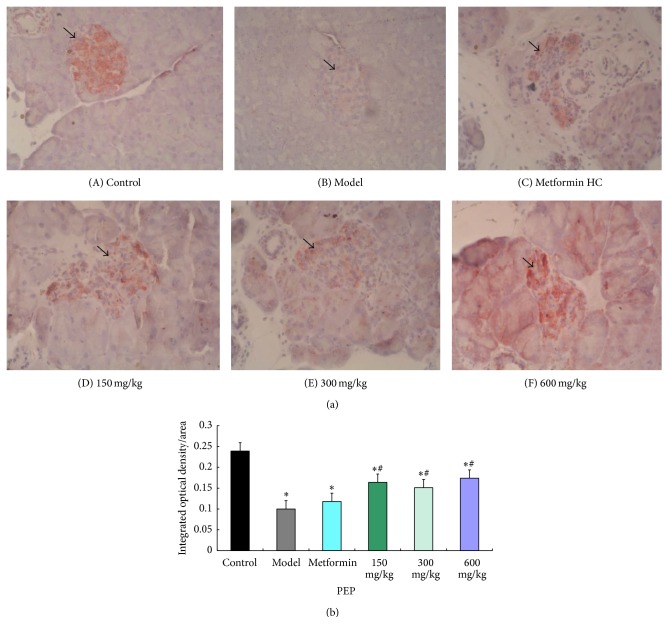
The effect of PEP on the histopathological structure of the pancreas in DM rats (IHC). A DM model was established by feeding rats a high-fat diet and injecting them with a low dose of STZ. After the model was established, PEP (0, 150, 300, and 600 mg/kg) was administered intragastrically as an intervention for 28 d (*n* = 10 rats). The pancreatic tissues were collected, embedded, and sectioned, and a specific antibody against insulin/proinsulin was utilized to observe the residual *β*-cells in islets by using immunohistochemical method (IHC). (A) control: control group; (B) model: model group; (C) metformin HC: metformin HC group; (D) 150 mg/kg: low-dose PEP group; (E) 300 mg/kg: medium-dose PEP group; (F) 600 mg/kg: high-dose PEP group. The arrow indicates islet tissue. (a) shows the histopathological structure of the pancreas in DM rats by IHC; (b) shows the mean integrated optical density of *β*-cells in islets in DM rats using the Image-Pro Plus 4.5 image analysis software.

The immunohistochemistry results indicated that, in the pancreatic islets, the positively stained cells containing cytoplasmic bright red granules were *β*-cells. In the control group, the islets were predominantly round or oval with different sizes, defined edges, and intact morphology; the islets were full of copious bright red granules, and the staining was dark. In the model group, the islet structure was damaged, the edges were irregular, the islet area was decreased, there were significantly fewer bright red granules, and the staining was light. Compared with the model group, islets with darker staining were present in the metformin HC group, and there were significantly more bright red granules; similar results were observed for the low-, medium-, and high-dose PEP groups (Figure 4(a)). The mean integrated optical density of *β*-cells in islets was significantly lower in the model group compared with the control group (*P* < 0.05). Compared with the model group, the mean integrated optical densities of *β*-cells were significant increased in the low-, medium-, and high-dose groups (*P* < 0.05). However, the differences between the model group and metformin HC group were not significant (Figure 4(b)).

### 3.7. The Effect of PEP on the mRNA Expression of GCK and InsR in Liver Tissue and of APN and GLUT-4 in Adipose Tissue in DM Rats

**Figure 5:**
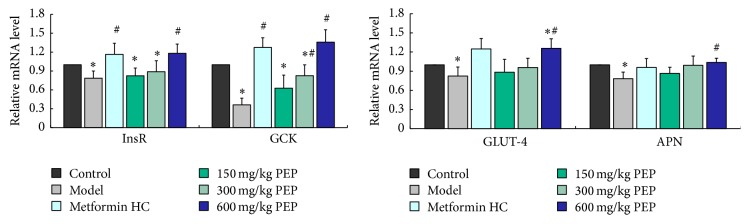
The effect of PEP on the mRNA expression of GCK and InsR in the liver and APN and GLUT-4 in adipose tissue in DM rats. A DM model was established by feeding rats a high-fat diet and injecting them with a low dose of STZ. After the model was established, PEP (0, 150, 300, and 600 mg/kg) was administered intragastrically as an intervention for 28 d. Subsequently, liver and adipose tissue was collected; mRNA was extracted, reverse transcribed into cDNA, and analyzed by real-time PCR to determine the mRNA expression levels in the rats in each group. The mRNA expression levels were determined using the relative quantification method (*β*-actin was used as the internal control). The data are presented as the mean ± standard deviation (*n* = 10 rats). Compared with the control group: ^*∗*^ *P* < 0.05; compared with the model group: ^#^ *P* < 0.05.

Compared with the model group, InsR mRNA expression in the liver in the low-, medium-, and high-dose PEP groups significantly increased (*P* < 0.05), and GCK mRNA expression in the liver in the low-, medium-, and high-dose PEP and the metformin HC groups increased (*P* < 0.05). APN mRNA expression in adipose tissue was higher in the high-dose PEP group than in the model group (*P* < 0.05). GLUT-4 mRNA expression in adipose tissue in the low-, medium-, and high-dose PEP and the metformin HC groups was not significantly different from that in the model group (*P* > 0.05), but GLUT-4 expression exhibited an increasing trend wherein the expression level increased with increasing PEP concentration (Figure 5).

## 4. Discussion

The etiology of DM is complex. It is primarily considered to result from a combination of genetic factors and various environmental factors [31]. There is no drug or method for the prevention or treatment of DM that has a significant therapeutic effect, few toxic side effects, and an acceptable safety profile [32–34]. Therefore, studying nutritious compounds that have minimal toxic side effects and can have long-term use is important for the prevention and treatment of DM.

Due to the different adsorption capacities of polysaccharides by the DEAE cellulose method and the different retention capabilities of polysaccharides by the Sephadex-100 gel method, we separated and highly purified polysaccharides from* Enteromorpha prolifera*. By infrared spectrum analysis, the main components of the polysaccharides were carbohydrates, which not only are rich in sulfate esters but also contain ether compounds and unsaturated carbon chains, which is in accord with previous studies [35]. The sulfate content of polysaccharides from* Enteromorpha prolifera* was 19.2% in our study, which was higher than that of previous studies [35]. Gas chromatographic analysis showed that the monosaccharides of PEP were rhamnose, glucuronic acid, arabinose, fucose, xylose, and glucose, which is inconsistent with a previous study in which galactose and mannose were observed [35]. The discrepancies in these results may be due to differences in the species, origin, and season of Enteromorpha.

High blood glucose levels, hyperinsulinemia (in the early stages), and low insulin sensitivity are the salient features of DM. Impaired glucose tolerance (IGT) occurs in the middle of the transition from normal to DM and is a primary feature of glucose metabolic disorders. The mechanism by which IGT occurs may be associated with IR and impaired islet *β*-cell function [36]. The rapid changes in glucose tolerance and/or blood sugar levels are prone to induce DM; therefore, OGTTs and blood glucose levels can be utilized to monitor and control the occurrence and development of DM [37]. This study confirmed that PEP reduced blood glucose levels in DM rats and significantly improved IGT with similar effects as the hypoglycemic agent metformin HC; in particular, prominent effects were observed in the medium-dose PEP group. Serum insulin levels were significantly reduced in the high-dose PEP group, and insulin sensitivity was significantly higher in all the PEP intervention groups. Furthermore, PEP intervention alleviated the diabetes symptom of drinking more water without changing caloric intake and inducing gut dysfunction for food consumption and body weight had no changed. These results indicated that PEP affected the regulation of glucose metabolism by improving IGT, promoting insulin secretion by islet *β*-cells, and enhancing insulin sensitivity.

High glucose can cause the nonenzymatic oxidation of proteins and induce oxidative stress [38]. Chronic exposure to high glucose levels increases the oxidation of glucose itself, which produces enediol and dihydroxy compounds as well as a significant amount of ROS [39]. Therefore, in the model used in the study, the high-glucose, high-fat diet that was fed to the rats stimulated oxidative stress; moreover, in combination with the STZ injection that damaged the pancreas, the oxidative damage in the pancreas as well as liver was exacerbated, resulting in DM. SOD is a key intracellular antioxidant that removes superoxide anion radicals in the body [40]. GSH-Px is a scavenger that protects the structure and functional integrity of the cell membrane by reducing hydrogen peroxide and lipid hydroperoxides [41]. MDA levels reflect the degree of lipid peroxidation in the body and indirectly reflect the degree of cellular damage [42]. In this study, compared with the control group, MDA levels increased, GSH-Px activity decreased, and SOD activity exhibited a decreasing trend in the model group, indicating that there was more severe oxidative damage in the DM rats, potentially attributable to the oxidative stress induced by the high-sugar, high-fat diet and STZ. After four weeks of PEP intervention, MDA levels significantly decreased and SOD and GSH-Px activities increased in a dose-dependent manner in all the PEP treatment groups compared with the model group. These results indicated that PEP inhibited lipid peroxidation in the cell membrane, relieved cellular oxidative damage, and promoted the functional recovery of tissues by enhancing antioxidant activity and promoting the clearance of free radicals and peroxidation products, such as MDA.

The pathological examination of the pancreas revealed that the number of pancreatic islet cells in the rats in the high- and medium-dose PEP groups significantly increased compared with the model group, whereas low-dose PEP had a slightly weaker effect. The cellular morphology of the pancreatic islet cells was nearly normal in the rats in the high- and medium-dose PEP groups. This result confirmed that PEP facilitated the repair of tissue damage and exerted a protective function in the pancreas.

It is currently understood that insulin acts by interacting with the InsR on the cell membrane (predominantly in hepatocytes, adipocytes, and muscle cells) and that its functions rely on receptor density and affinity in the cell membrane. Therefore, InsR is the first key point of action for insulin, and the InsR gene is critical for studies of IR [43]. Our study demonstrated that mRNA level of InsR was significantly higher in the low-, medium-, and high-dose PEP groups than that in the model group; this result was consistent with the majority of previous studies that have reported a reduction in both the number and affinity for insulin of membrane InsR in liver and muscle tissue of DM rats with IR [44]. The blood sugar and insulin levels in the model group remained high, indicating that PEP reversed IR by upregulating the number of InsR. Further studies are needed to elucidate the precise mechanisms.

GCK is a key enzyme in the regulation of glucose metabolism and insulin secretion and is the first rate-limiting enzyme in glycolysis. It catalyzes the phosphorylation of glucose to glucose-6-phosphate, and this process is a prerequisite for liver glycogen synthesis. The functional loss or decreased expression of GCK suppresses glucose utilization in the liver and decreases insulin secretion from islet *β*-cells. Studies have reported that GCK is critical for beta cell hyperplasia under the condition of high-fat diet-induced IR [45]. Therefore, mutations in the GCK gene may be a cause of DM. Conversely, enhancing GCK gene expression may have a therapeutic effect on DM. Our study demonstrated that significantly higher GCK mRNA expression levels in the livers of rats in the low-, medium-, and high-dose PEP groups compared with those in the model group; the difference of GCK mRNA expression levels between the high-dose group and the metformin HC group was most dramatic. Previous study showed that dietary phenolic compounds altered glucose metabolism and decreased the risk of type 2 diabetes by increasing the level of glucokinase mRNA [46]. Therefore, it is possible that PEP improved glucose metabolism in DM rats by upregulating GCK mRNA expression.

GLUT-4 is a glycoprotein of 509 amino acids that is highly expressed in insulin-sensitive tissues, including muscle, adipose, and liver. These tissues also have high levels of glucose uptake and utilization; these processes are regulated by blood glucose and insulin [47]. Transmembrane glucose transport is mediated by GLUT-4. Under resting conditions, 90% of GLUT-4 molecules are distributed on the inner cell membrane, and only a small portion is on the outer cell membrane. When the insulin stimulation signal is transmitted into cells, GLUT-4 translocates from the inner to the outer membrane, therefore increasing glucose transport [48]. When GLUT-4 function is impaired, glucose uptake as well as utilization in peripheral tissues decreases, particularly in skeletal muscle and adipose tissue, leading to postreceptor IR. Therefore, GLUT-4 dysfunction is a major contributor to the onset of DM [49]. This study determined that GLUT-4 mRNA expression in adipose tissue decreased in the rat models; despite the fact that the differences between the intervention groups and the model group were not significant, PEP intervention induced GLUT-4 mRNA expression to various extents. Previous study showed that the alterations in GLUT-4 mRNA level in adipose tissue contributed to the physiological activities of n-3 PUFA in preventing body fat accumulation and in regulating glucose metabolism in rats [50]. Therefore, it is possible that PEP improved glucose metabolism by upregulating the GLUT-4 mRNA expression.

APN, also termed Acrip30/AMP1/GBP28, is a cytokine that is secreted by adipocytes. APN is composed of 247 amino acids; the APN gene is located on 3q27 and encodes susceptibility genes for DM and metabolic syndrome [51]. APN is a protective factor for IR, and therefore it is highly correlated with insulin sensitivity and plays an important role in IR. Similar to insulin, APN promotes glucose and fat utilization and energy consumption in muscle and inhibits gluconeogenesis and the synthesis of fatty acids and cholesterol in the liver [52]. APN is predominantly expressed in adipocytes, and the APN gene is only expressed in white adipose tissue; therefore, changes in adipocyte number can affect APN expression. It has been demonstrated in animal models that injecting APN into lipoatrophic mice partially reversed IR [53] and that the ability to control blood sugar levels with insulin in transgenic rats with high APN expression significantly increased [54]. In humans, the direct injection of APN reduces blood sugar levels [55], and increased APN increases insulin sensitivity in obese, DM, and IR patients. Therefore, adipocytokines, such as APN, reach target organs via various pathways to induce IR-related diseases, and the downregulation of related factors might prevent IR by improving insulin sensitivity. In this study, we determined that the difference in APN gene expression between the high-dose PEP group and the model group was significant, whereas APN gene expression was higher in the low- and medium-dose PEP groups than in the model group but did not reach significance. These results indicated that PEP improved IR and enhanced insulin sensitivity by upregulating APN gene expression in rat white adipose tissue and that it therefore has potential for the prevention of DM.

Previous study had showed that* Enteromorpha compressa* and polysaccharide fraction from* Enteromorpha prolifera* exhibit potent antioxidant activity [17, 18]. These studies indicate that PEP might directly exhibit antioxidant activity to alleviate oxidative damage in the pancreas, and finally improve glucose metabolism. However, PEP might improve glucose metabolism by upregulating the InsR, GCK, APN, and GLUT-4 mRNA expression in our present study, which may alleviate oxidative damage for high glucose, ultimately result in nonenzymatic oxidation of proteins, and induce oxidative stress [38]. Therefore, in our present study, it is unclear whether the improvements in glucose drive the improvements in oxidative measures or vice versa. Further work, especially in vitro study, in this area is warranted.

## 5. Conclusion

Our results indicate that PEP can improve glucose metabolism in DM rats. The mechanism may relate to the antioxidant activity of PEP and its ability to regulate the mRNA level of InsR, GCK, APN, and GLUT-4 gene in liver and adipose tissue. These findings may suggest that PEP could be useful for the therapy of diabetes mellitus.

## Figures and Tables

**Table 1:** The effect of PEP on body weight, food consumption, and drinking water intake in DM rats.

Group	Body weight (g)	Food consumption (g/day/dam)	Drinking water intake (mL/day/dam)
Control	517.75 ± 29.57^*∗*^	11.26 ± 2.05^*∗*^	15.21 ± 1.71^*∗*^
Model	359.12 ± 57.69^#^	9.85 ± 1.38^#^	34.18 ± 5.03^#^
Metformin HC	354.73 ± 58.61^#^	9.29 ± 1.71^#^	30.79 ± 5.83^#^
150 mg/kg PEP	382.20 ± 40.36^#^	9.37 ± 2.26^#^	28.96 ± 8.39^#^
300 mg/kg PEP	375.93 ± 43.58^#^	8.98 ± 1.63^#^	24.68 ± 5.29^*∗*#^
600 mg/kg PEP	358.60 ± 35.08^#^	8.83 ± 1.69^#^	16.61 ± 2.82^*∗*^

A DM model was established by feeding rats a high-fat diet and injecting them with a low dose of STZ. After the model was established, PEP (150, 300, and 600 mg/kg) was administered intragastrically for 28 d. The body weight, food consumption, and drinking water intake were measured. The data are presented as the mean ± standard deviation (*n* = 10 rats). Compared with the model group: ^*∗*^ *P* < 0.05; compared with the control group: ^#^ *P* < 0.05.

**Table 2:** The effect of PEP on FBG levels in DM rats.

Group	FBG (mg/dL)				
	Day 0	Day 7	Day 14	Day 21	Day 28
Control	155.34 ± 35.1^*∗*^	176.76 ± 38.52^*∗*^	161.64 ± 27.18^*∗*^	161.10 ± 29.88^*∗*#^	138.78 ± 28.62^*∗*^
Model	321.48 ± 46.1^#^	258.48 ± 35.82^#^	248.76 ± 39.60^#^	283.68 ± 37.98^#^	258.12 ± 58.68^#^
Metformin HC	328.86 ± 14.58^*∗*^	148.32 ± 15.66^*∗*^	151.38 ± 16.38^*∗*#^	153.18 ± 16.38^*∗*#^	150.48 ± 19.08^*∗*#&^
150 mg/kg PEP	297.36 ± 40.86^#^	180.72 ± 19.62^*∗*^	167.76 ± 41.40^*∗*^	194.76 ± 34.02^*∗*#^	162.00 ± 33.30^*∗*#&^
300 mg/kg PEP	291.42 ± 36.54^#^	159.48 ± 28.26^*∗*^	152.28 ± 36.90^*∗*^	172.08 ± 38.88^*∗*^	149.04 ± 34.56^*∗*&^
600 mg/kg PEP	325.08 ± 44.64^#^	175.14 ± 26.10^*∗*^	160.74 ± 29.16^*∗*^	173.34 ± 31.32^*∗*^	158.76 ± 37.80^*∗*&^

A DM model was established by feeding rats a high-fat diet and injecting them with a low dose of STZ. After the model was established, PEP (150, 300, and 600 mg/kg) was administered intragastrically for 28 d. Blood samples from the caudal vein were collected to determine FBG levels using a blood glucose meter. The data are presented as the mean ± standard deviation (*n* = 10 rats). Compared with the model group: ^*∗*^ *P* < 0.05; compared with the control group: ^#^ *P* < 0.05; compared with day 0: ^&^ *P* < 0.05.

**Table 3:** The effect of PEP on FINS levels and the ISI in DM rats.

Group	FINS (mU/L)	ISI
Control	3.27 ± 0.20^*∗*^	−3.05 ± 0.30^*∗*^
Model	3.63 ± 0.25^#^	−3.87 ± 0.32^#^
Metformin HC	3.39 ± 0.27^*∗*^	−2.92 ± 0.13^*∗*^
150 mg/kg PEP	3.55 ± 0.29	−3.33 ± 0.32^*∗*#^
300 mg/kg PEP	3.59 ± 0.15	−3.18 ± 0.31^*∗*^
600 mg/kg PEP	3.39 ± 0.23^*∗*^	−3.21 ± 0.44^*∗*^

A DM model was established by feeding rats a high-fat diet and injecting them with a low dose of STZ. After the model was established, PEP (150, 300, and 600 mg/kg) was administered intragastrically as an intervention for 28 d. Blood samples from angular vein were collected to measure the FBG and FINS levels with the ELISA kit, and the ISI was calculated. The data are presented as the mean ± standard deviation (*n* = 10 rats). Compared with the model group: ^*∗*^ *P* < 0.05; compared with the control group: ^#^ *P* < 0.05.

**Table 4:** The effect of PEP on MDA, SOD, and GSH-Px in DM rats.

Group	MDA (nmol/mL)	SOD (U/mL)	GSH-Px (*μ*mol/L)
Control	5.09 ± 0.63^*∗*^	252.68 ± 24.92^*∗*^	4800.00 ± 449.46^*∗*^
Model	5.94 ± 0.89^#^	243.45 ± 19.32	3680.00 ± 388.65^#^
Metformin HC	5.05 ± 0.51^*∗*^	239.90 ± 28.09	4164.57 ± 589.16^*∗*#^
150 mg/kg PEP	5.03 ± 0.45^*∗*^	271.31 ± 23.37^*∗*^	3979.43 ± 397.13^#^
300 mg/kg PEP	4.25 ± 0.67^*∗*#^	272.63 ± 22.25^*∗*^	4132.57 ± 483.74^*∗*#^
600 mg/kg PEP	4.56 ± 0.96^*∗*^	269.09 ± 26.05^*∗*^	4304.00 ± 341.72^*∗*#^

A DM model was established by feeding rats a high-fat diet and injecting them with a low dose of STZ. After the model was established, PEP (150, 300, and 600 mg/kg) was administered intragastrically as an intervention for 28 d. Blood samples from angular vein were collected to quantitate the enzymatic activity of MDA (nmol/mL), SOD (U/mL), and GSH-Px (*μ*mol/L) in serum. The data are presented as the mean ± standard deviation (*n* = 10 rats). Compared with the model group: ^*∗*^ *P* < 0.05; compared with the control group: ^#^ *P* < 0.05.

## Abbreviations

PEP: Polysaccharides from* Enteromorpha prolifera*
DM: Diabetes mellitus
STZ: Streptozotocin
FBG: Fasting blood glucose
INS: Insulin
ISI: Insulin sensitivity index
GCK: Glucokinase
InsR: Insulin receptor
GLUT-4: Glucose transporter type 4
APN: Adiponectin
IR: Insulin resistance
SOD: Superoxide dismutase
GSH-Px: Glutathione peroxidase
MDA: Malondialdehyde
RT-PCR: Reverse transcription polymerase chain reaction
OGTT: Oral glucose tolerance test
ROS: Reactive oxygen species.
